# Eculizumab in treatment for complement-mediated thrombotic microangiopathy associated with acute pancreatitis

**DOI:** 10.17179/excli2025-8881

**Published:** 2025-12-18

**Authors:** Shruti Shettigar, Rutvikkumar Jadvani, Dwij Doshi, Chintan V. Shah

**Affiliations:** 1 Division of Nephrology, Hypertension, and Renal Transplantation, University of Florida -College of Medicine, Gainesville, Florida, USA; 2 Rahul International School, Mira Road, Thane, Maharashtra, India

## ⁯

Thrombotic microangiopathy (TMA) is a pathological syndrome characterized by clinical features including microangiopathic hemolytic anemia, thrombocytopenia, and organ injury (George and Nester, 2014[1]), which may or may not be associated with accelerated activation of the alternate complement pathway. In the past, cases of secondary TMAs have been reported in which complement activation occurs due to underlying diseases. However, there is scarce evidence about the efficacy of complement-targeted therapies, with ongoing clinical trials testing complement inhibitors in secondary TMAs (Swisher et al., 2007[3]). We describe a case of complement-mediated TMA (CM-TMA) secondary to acute alcohol-induced pancreatitis, successfully treated with eculizumab. This case highlights the role of complement blockade in secondary forms of CM-TMA, especially when precipitated by acute pancreatitis. 

A 53-year-old man with chronic alcohol use (10-12 beers daily) and gout presented with acute epigastric abdominal pain, nausea, and vomiting. He was diagnosed with acute pancreatitis with a serum lipase level of 3088 U/L and a CT scan of the abdomen and pelvis demonstrating acute interstitial pancreatitis with peripancreatic inflammatory stranding. Over the next four days, although pancreatitis-related symptoms resolved, the patient developed significant thrombocytopenia with platelets declining from 230,000 cells/uL to 22,000 cells/uL, hemolytic anemia with hemoglobin declining from 11.8 gm/dl to 4.7 gm/dl, high lactate dehydrogenase (LDH) of 2549 U/L, undetectably low haptoglobin (less than 30 mg/dL) and elevated indirect bilirubin of 4.7 mg/dl. A peripheral blood smear demonstrated 10-15 schistocytes per high-power field, suggesting microangiopathic hemolytic anemia (MAHA). The patient also developed acute kidney injury (AKI) with a creatinine of 2.8 mg/dL from a baseline of 0.8mg/dl with a 24-hour urinary protein of 7.7 grams.

Although the PLASMIC score was high, plasma exchange was not offered, as ADAMTS13 activity by ELISA was 67.5 U/dL (normal < 10 U/dL), making TTP less likely. Stool testing was negative for Shiga toxin-producing E. coli (STEC). Genetic testing for complement pathway disorders (Mayo Clinic Laboratory, Inc.) was negative. Further testing for functional assessment of the alternate complement pathway revealed significantly elevated SC5b-9 at 534 ng/ml (normal <251 ng/ml), and Factor Bb >6 mcg/ml (normal <1.7 mcg/ml), indicating accelerated activation of the alternate complement pathway, likely due to acute pancreatitis. 

A decision was made to initiate targeted therapy against C5 with Eculizumab. A single dose of Eculizumab 900 mg IV was given along with appropriate prophylactic vaccination for meningococcus and antibiotic coverage with penicillin V. Within 7 days following Eculizumab, platelet counts improved to 445,000 cells/uL, serum LDH levels improved to 550 IU/L, haptoglobin increased to 86 mg/dl, hemoglobin stabilized around 7.8 g/dL and serum creatinine level improved to 1.2 mg/dL. The serial changes in LDH, platelet count, haptoglobin, and serum creatinine after a single dose of Eculizumab (900 mg IV) are shown in the Supplementary information, Figure 1. At 2 months of follow-up, the patient remained in hematological and renal remission without any further need for complement inhibitor therapy.

Complement-mediated endothelial damage has been implicated in secondary forms of TMA, including hypertensive urgency, pregnancy-associated, and transplant-associated TMA (Morelle et al., 2025[2]), but the role of the alternate complement pathway blockade has not been thoroughly studied. During the pre-eculizumab era, Swisher et al. reported 5 cases of TMA (TTP-HUS-like syndrome) preceded by acute pancreatitis and treated temporarily with plasma exchange, without relapse on long-term follow-up (Swisher et al., 2007[3]). Notably, only 2 of the 5 patients had low ADAMTS13 activity, suggestive of TTP. Additionally, alternative complement pathway activity was not assessed in those cases; therefore, it is unclear whether it was activated and if complement inhibitors could have offered any therapeutic benefit. While multiple clinical trials are currently underway to examine the role of complement pathway inhibitors in secondary TMA (Swisher et al., 2007[3]), we demonstrated that a transient activation of the alternate complement pathway might be the underlying etiology of TMA in some cases associated with pancreatitis, and short-term complement inhibition with eculizumab can offer non-invasive, safer and efficacious therapy option with a rapid and dramatic response.

## Declaration

### Conflict of interest

The authors have no conflict of interest to declare.

### Artificial Intelligence (AI) - assisted technology

The authors declare they have not used AI in the writing and production of the present manuscript.

## Supplementary Material

Supplementary information
