# Supplementary material for: Eculizumab in treatment for complement-mediated thrombotic microangiopathy associated with acute pancreatitis
Source: EXCLI J. 2025 Dec 18;24:1802–3. doi: 10.17179/excli2025-8881 (PMC12853028; doi:10.17179/excli2025-8881)
Supplement: Supplementary information [file EXCLI-24-1802-s-001.pdf]

EXCLI Journal 2025;24:1802-1803 – ISSN 1611-2156
Received: September 01, 2025, accepted: November 15, 2025, published: December 18, 2025

Supplementary information to:

Letter to the editor:

ECULIZUMAB IN TREATMENT FOR COMPLEMENT-MEDIATED THROMBOTIC MICROANGIOPATHY ASSOCIATED WITH ACUTE PANCREATITIS

Shruti Shettigar[1], Rutvikkumar Jadvani[1], Dwij Doshi[2], Chintan V. Shah[1]

[1] Division of Nephrology, Hypertension, and Renal Transplantation, University of Florida - College of Medicine, Gainesville, Florida, USA
[2] Rahul International School, Mira Road, Thane, Maharashtra, India

* **Corresponding author:** Chintan V. Shah, M.D., Associate Professor of Medicine, Division of Nephrology, Hypertension, and Renal Transplantation, University of Florida - College of Medicine, 1600 SW Archer Road, Room CG-98, Gainesville, FL 32610, Office: (352) 294-8790. E-mail: shahc@ufl.edu

https://dx.doi.org/10.17179/excli2025-8881

This is an Open Access article distributed under the terms of the Creative Commons Attribution License (https://creativecommons.org/licenses/by/4.0/).

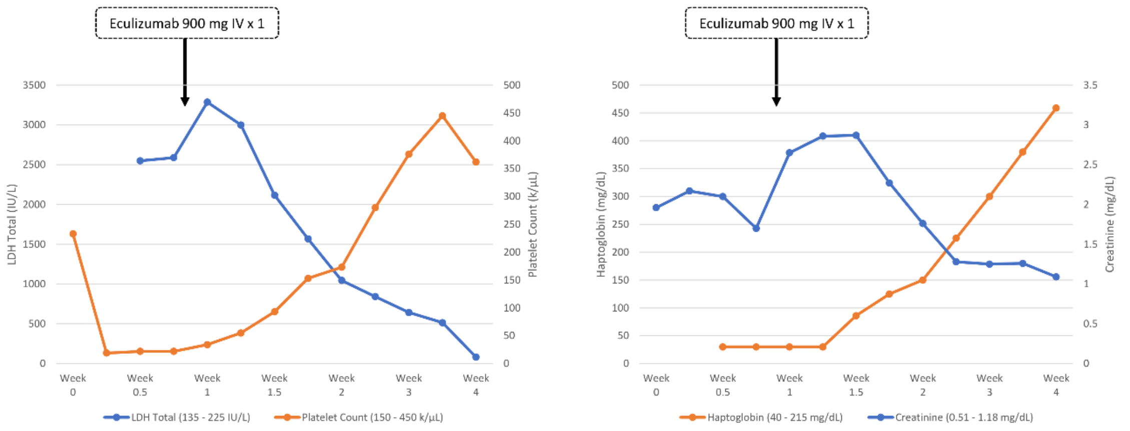

Supplementary Figure 1: The serial changes in LDH, platelet count, haptoglobin, and serum creatinine after a single dose of Eculizumab (900 mg IV)
